# Role of Symbiotic Auxotrophy in the *Rhizobium*-Legume Symbioses

**DOI:** 10.1371/journal.pone.0013933

**Published:** 2010-11-11

**Authors:** Jurgen Prell, Alexandre Bourdès, Shalini Kumar, Emma Lodwig, Arthur Hosie, Seonag Kinghorn, James White, Philip Poole

**Affiliations:** 1 John Innes Centre, Norwich Research Park, Norwich, United Kingdom; 2 School of Biological Sciences, University of Reading, Reading, United Kingdom; Newcastle University, United Kingdom

## Abstract

**Background:**

*Rhizobium leguminosarum* bv. *viciae* mutants unable to transport branched-chain amino acids via the two main amino acid ABC transport complexes AapJQMP and BraDEFGC produce a nitrogen starvation phenotype when inoculated on pea (*Pisum sativum*) plants [1], [2]. Bacteroids in indeterminate pea nodules have reduced abundance and a lower chromosome number. They reduce transcription of pathways for branched-chain amino acid biosynthesis and become dependent on their provision by the host. This has been called “symbiotic auxotrophy”.

**Methodology/Principal Findings:**

A region important in solute specificity was identified in AapQ and changing P144D in this region reduced branched-chain amino acid transport to a very low rate. Strains carrying P144D were still fully effective for N_2_ fixation on peas demonstrating that a low rate of branched amino acid transport in *R. leguminosarum* bv. *viciae* supports wild-type rates of nitrogen fixation. The importance of branched-chain amino acid transport was then examined in other legume-*Rhizobium* symbioses. An *aap bra* mutant of *R. leguminosarum* bv. *phaseoli* also showed nitrogen starvation symptoms when inoculated on French bean (*Phaseolus vulgaris*), a plant producing determinate nodules. The phenotype is different from that observed on pea and is accompanied by reduced nodule numbers and nitrogen fixation per nodule. However, an *aap bra* double mutant of *Sinorhizobium meliloti* 2011 showed no phenotype on alfalfa (*Medicago sativa*).

**Conclusions/Significance:**

Symbiotic auxotrophy occurs in both determinate pea and indeterminate bean nodules demonstrating its importance for bacteroid formation and nodule function in legumes with different developmental programmes. However, only small quantities of branched chain amino acids are needed and symbiotic auxotrophy did not occur in the *Sinorhizobium meliloti-*alfalfa symbiosis under the conditions measured. The contrasting symbiotic phenotypes of *aap bra* mutants inoculated on different legumes probably reflects altered timing of amino acid availability, development of symbiotic auxotrophy and nodule developmental programmes.

## Introduction

The largest input of available nitrogen into the biosphere comes from biological reduction of atmospheric N_2_ to ammonium [3], with legume-*Rhizobium* symbioses responsible for much of this. These symbioses arise from infection of host plants, mainly of the legume family, by proteobacteria and results in root structures called nodules [4]. The symbiosis is initiated by flavonoids released from the plant roots which in return elicit the synthesis of lipochitooligosaccharide Nod factors by the rhizobial partner. Bacterial cells are trapped by curling root hairs and proliferate in an infection focus. This turns into an infection thread that grows into the root cortex where a zone of meristematic cells is newly induced. Here the bacteria are released from the infection threads into the plant cells by endocytosis and surrounded by a plant derived symbiosome membrane [5]. The whole structure develops into a nodule with plant cells filled with thousands of symbiosomes. Nodules can be classified as determinate or indeterminate, with those in the Inverted-Repeat-Lacking Clade (IRLC) of the legume subfamily *Papilionoideae* (such as *Medicago, Pisum* or *Vicia*) being indeterminate with a cylindrical shape and a persistent meristem at the tip of the nodule. Bacteria inside symbiosomes differentiate into N_2_ fixing bacteroids and this is accompanied by dramatic increases in size, shape and DNA content [6]. The differentiation is caused by a family of plant peptides that share similarities with antimicrobial peptides and probably interfere with the bacterial cell cycle [7]. Nodules of the Milletioides clade (such as *Phaseolus, Vigna* or *Glycine*), form spherical determinate nodules with a transient meristem; bacteria do not undergo endoreduplication and therefore do not enlarge substantially. These bacteroids retain a normal DNA content and are largely viable after isolation from nodules [6]. However, other combinations also exist, including enlarged bacteroids in determinate nodules and non-enlarged bacteroids in indeterminate nodules, with the evolutionary newer form of enlarged bacteroids evolving at least 5 times independently in the legume family [8].

The shared metabolism between the symbiotic partners has been proposed to be a simple nutrient exchange of dicarboxylates for ammonium in the nodule [9], [10], [11], but amino acid transport by bacteroids has also been shown to be essential in pea (*Pisum sativum*) nodules [1]. *R. leguminosarum* bv. *viciae* strains contain two broad specificity amino acid ABC (ATP binding cassette) transporters, AapJQMP and BraDEFGC. AapJQMP consists of a solute binding protein (SBP) AapJ, two permease units AapQM and an ABC subunit dimer formed by AapP for ATP hydrolysis. The BraDEFGC complex consists of SBP BraC, permeases BraDE and ABC subunits BraFG. The *aap bra* double mutants, RU1357 and RU1722, formed pea bacteroids that appeared morphologically normal in electron micrographs and fixed nitrogen per bacteroid at wild-type levels, but the plants were nitrogen starved [1]. However, *aap bra* mutant bacteroid protein levels per plant were reduced to ∼30% of wild-type. The reason for the nitrogen starved phenotype was later identified to be a limitation of branched-chain amino acid biosynthesis by developing bacteroids [2]. Branched-chain amino acid biosynthesis is transcriptionally reduced during bacteroid development and results in symbiotic auxotrophy where amino acids must be supplied by the plant.

Here we provide evidence that only a low rate of branched-chain amino acid transport is required to overcome symbiotic auxotrophy of *R. leguminosarum* bv. *viciae* strains in pea nodules. This shows that large quantities of amino acid are not needed and is consistent with symbiotic auxotrophy. Additionally we report the symbiotic phenotypes of an *aap bra* mutant in *R. leguminosarum* bv. *phaseoli* inoculated on French bean (*Phaseolus vulgaris*) forming determinate nodules and *Sinorhizobium meliloti* on alfalfa (*Medicago sativa*) forming indeterminate nodules.

## Results

### AapQ site directed mutants with altered transport rates

Previous bioinformatic analysis suggested that a conserved N-terminal domain of the polar amino acid transporting permease proteins (PAAT family of ABC transporters) such as AapQ and AapM might differentially affect the uptake of amino acids [12]. Residue 53 of this domain (residue 144 in AapQ) is strongly correlated with the charge of the transported solute. Thus transporters of basic amino acids have asp or glu at residue 53, while acid transporters have a branched-chain amino acid and broad specificity transporters (e.g. AapQ) have proline. In order to investigate this, a number of site directed mutants were isolated in AapQ and screened for transport specificity by expression from a plasmid in the *aap bra* null mutant RU1722 (data not shown). Of these mutants P144D proved most interesting, so a single copy of AapQ P144D was generated by recombination into the chromosome of Rlv3841. This was achieved by replacing the ΩSp cartridge in RU1722 *aapJQM*::ΩSp *braEF*::ΩTc with a full copy of the *aapJQM* operon containing the mutated AapQ P144D, generating RU1976. The *braEF*::ΩTc null mutation is present in all the control strains so transport occurs exclusively via Aap. The uptake properties of RU1976 (*aapQ* P144D *braEF*::ΩTc) were compared to those of RU1721 (*braEF*::ΩTc), which contains a wild-type copy of Aap and the *aap bra* deletion mutant RU1722 (*aapJQM*::ΩSp *braEF*::ΩTc) (Table 1). The maximum rate (V_max_) of transport of small amino acids such as alanine was less severely affected (51% of control) by the AapQ P144D mutation than transport of larger amino acids such as glutamate (25% of control). The lower levels of amino acid uptake observed in RU1976 could also arise from a reduction in solute affinity (K_m_). In order to investigate this, uptake rates were determined for glutamate, alanine, aspartate and leucine at varying concentrations in both RU1721 and RU1976. These were then used to calculate K_m_ for amino acid uptake by these strains (Table 1). It is clear that the reduction in amino acid uptake in RU1976 is not due to reduced affinity of Aap for its solutes, indeed, the K_m_ of AapQ P144D is slightly lower than wild-type. Rather, in each case, V_max_ is lower in RU1976. Similar drops in transport rates were determined for all branched-chain amino acids (Table 2).

**Table 1:** Transport of amino acids by strains of *R. leguminosarum* bv. *viciae.*

Strain	Genotype	GlutamateVmax(Km)	AlanineVmax(Km)	AspartateVmax(Km)	LeucineVmax(Km)
RU1721	*braEF*::ΩTc	9.80±0.30(106.3±11.8)	10.44±0.15(215.5±89.7)	6.75±0.75(75.7±12.4)	4.71±0.14(391.7±76.49)
RU1976	*aapQ* P144D *braEF*::ΩTc	2.42±0.28(36.0±3.02)	5.32±0.76(91.0±1.0)	3.22±0.50(53.0±3.7)	1.37±0.17(279.0±80.5)
RU1722	*aapJQM*::ΩSp *braEF*::ΩTc	0.2±0.1(ND)	3.3±0.1(ND)	0.2±0.1(ND)	0.8±0.1(ND)

Vmax and Km were calculated from transport rate mean values of 3 independent cultures measured at 5 concentrations from 0.1 µM up to 5 µM. Vmax values are given in nmol min^−1^mg^−1^ protein ± SEM and Km mean values are given in nM ± SEM. ND  =  not determined.

**Table 2:** Transport rates of branched-chain amino acids and plant dry weights by strains of *R. leguminosarum* bv. *viciae.*

Strain	Genotype	Alanine	Leucine	Isoleucine	Valine	Plant dry weights
RU1721	*braEF*::ΩTc	10.4±1.6	3.9±0.4	3.0±0.2	4.0±0.1	1.14±0.08
RU1976	*aapQ* P144D*braEF*::ΩTc	7.0±1.1	1.5±0.1	1.1±0.1	2.0±0.2	1.12±0.08
RU1722	*aapJQM*::ΩSp*braEF*::ΩTc	2.8±0.2	0.6±0.1	0.6±0.3	0.3±0.1	0.65±0.04

Transport rates are the mean ± SEM of 3 independent cultures expressed as nmol min^−1^mg^−1^ protein. Dry weights (g plant^−1^) are averages of 15 plants.

### Plant phenotypes of P144D mutants on peas

In order to analyse if reduced transport by Aap triggers symbiotic auxotrophy, strain RU1976 and the control strains, RU1721 and RU1722 were inoculated onto pea plants. Dry weights of 6 week old pea plants inoculated with RU1976 and RU1721 were similar, while RU1722 dry weights were reduced as expected (Table 2). Thus a ∼60% reduction in the rate of branched-chain amino acid uptake in strain RU1976 compared to RU1721, did not significantly alter N_2_-fixation and presumably bacteroid formation. Consistent with the proposal of symbiotic auxotrophy only a low rate of branched-chain amino acid transport is needed for N_2_-fixation.

### Transport phenotype of an *aap bra* mutant in *R. leguminosarum* bv. *phaseoli*

The transport specificities and symbiotic phenotypes of *R. leguminosarum* bv. *viciae* strain A34 and its *aapJQM*::ΩSp *braE*::Tn*phoA* mutant RU1357, have been previously described [1], [13]. *R. leguminosarum* bv. *phaseoli* 4292 and *R. leguminosarum* bv. *viciae* A34 are isogenic strains that only carry different symbiotic (sym) plasmids. Strain 4292 nodulates *Phaseolus* while strain A34 nodulates *Pisum*, *Vicia* and *Lens* [14]. Mutants, *aapJQM*::ΩSp (RU1809), *braE::*Tn*phoA* (RU1932) and *aapJQM*::ΩSp *braE::*Tn*phoA* (RU1933) were isolated in *R. leguminosarum* bv. *phaseoli* 4292 as described in Materials and Methods. Transport of several amino acids by these strains confirmed that Aap and Bra have indistinguishable properties in *R. leguminosarum* bv *viciae* A34 and *R. leguminosarum* bv. *phaseoli* 4292 (Table 3; compare to [13]). Thus in strain 4292 glutamate transport is mainly facilitated via Aap while the branched-chain amino acid leucine is primarily transported via Bra. α-Aminoisobutyric acid (AIB), alanine and histidine are transported at similar rates by both transporters and GABA is exclusively transported via Bra. In the *aap bra* double mutant (RU1933) the transport of all solutes was reduced to background levels, except for alanine and histidine which under the assay conditions can still enter the cells via other transport systems [13]. It is important to note here, that branched-chain amino acid transport is abolished, which is essential to explain the previously reported phenotype of *R. leguminosarum* bv. *viciae* double transport mutants on pea plants [1], [2]. *R. leguminosarum* bv. *viciae* cells are dependent on the provision of branched-chain amino acids by the host plant to develop into mature bacteroids and probably also for their persistence [2].

**Table 3:** Transport of amino acids and plant dry weights by strains of *R. leguminosarum* bv *phaseoli.*

Strain	Genotype	Glutamate	AIB	Alanine	Histidine	GABA	Leucine	Plant dry weights
RU2222	Wild-type	27.4±0.2	25.0±1.2	36.4±1.9	7.0±0.6	11.1±0.6	10.4±0.8	1.37±0.08
RU1809	*aapJQM*::ΩSp	7.9±0.2	13.3±0.3	16.5±0.4	3.8±0.4	10.3±1.1	7.8±0.2	1.28±0.06
RU1932	*braE*::Tn*phoA*	22.4±0.5	11.5±1.6	25.6±2.5	5.5±0.1	<0.1	4.9±0.4	1.44±0.05
RU1933	*aapJQM*::ΩSp*braE*::Tn*phoA*	0.5±0.1	<0.1	5.6±0.5	1.7±0.23	<0.1	0.4±0.3	1.04±0.05

All strains were grown on AMS glucose/NH_4_Cl minimal medium. Rates of uptake are expressed as nmol min^−1^mg^−1^ protein. Values are the mean ± SEM of determinations from three or more independent cultures. Dry weights (g plant^−1^) are averages of >27 plants.

### Plant phenotypes of *aap bra* mutants on beans

**Figure 1:**
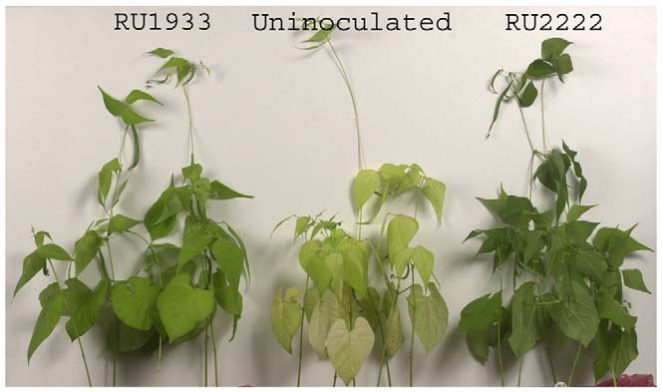
Growth of beans infected by different strains of *R. leguminosarum* bv. *phaseoli*. Plants were inoculated with RU2222 (wild-type), RU1933 (*aapJQM*::ΩSp *braE*::Tn*phoA*) or left uninoculated.

Growth and symbiotic performance of French bean (*P. vulgaris*) was measured at different time points. Plants at 8 weeks showed no significant differences when inoculated with the wild-type (RU2222), the *aap* mutant (RU1809) and the *bra* mutant (RU1932) (Table 3). However, the *aap bra* double mutant, RU1933, produced significantly (p<0.001) reduced dry weights compared to wild-type RU2222 inoculated plants. Plants inoculated with RU1933 also showed signs of yellowing after 8 weeks although not as severe as uninoculated controls (Figure 1). Nitrogen fixation rates, measured as acetylene reduction (AR), and nodule numbers were determined after 4, 5 and 6 weeks of plant growth. Because bacteroid protein levels per pea plant were reduced in *aap bra* mutants of *R. leguminosarum* bv. *viciae* [1], [2] this was also measured in bean nodules after 4 weeks of growth. A strong reduction in nitrogen fixation rates (down to 36% of the wild-type control; Table 4) was accompanied by a reduced nodule number (down to 54%) after 4 weeks of plant growth (Table 4). In addition the level of acetylene reduction per nodule formed by RU1933 was significantly (p = 0.005) reduced after 4 weeks compared to levels in wild-type nodules (Table 4). Nitrogen fixation rates and nodule numbers were also markedly reduced on 5 and 6 weeks old plants inoculated with RU1933 compared to wild-type (Table 5), while acetylene reduction rates per nodule recovered. However, compared to 4 week old plants, nodule numbers on 5 and 6 week old plants were generally higher, which were also grown in bigger pots (see Materials and Methods).

**Table 4:** Symbiotic properties of 4 week old bean plants.

Strain	Genotype	AR(µmol h^−1^ plant^−1^)	Nodule number	Bacteroid protein per plant (mg)	Bacteroid protein per nodule (ug)	AR per nodule (nmol h^−1^)	AR per bac prot (µmol h^−1^ mg prot^−1^)
RU2222	wild-type	3.6±0.3	261±13	0.90±0.07	3.5±0.3	13.9±0.8	4.1±0.3
RU1933	*aapJQM*::ΩSp*braE*::Tn*phoA*	1.3±0.3	142±18	0.54±0.13	3.7±0.6	8.6±1.2	2.4±0.1
p-values		<0.001	<0.001	0.036	0.724	0.005	<0.001

The values are the mean ± SEM of plants inoculated with RU2222 (n = 8) and RU1933 (n = 6). P-values were determined by ttest.

**Table 5:** Symbiotic properties of 5 and 6 week old bean plants.

Strain	Genotype	AR(µmol h^−1^ plant^−1^)	Nodule number	AR per nodule (nmol h^−1^)	AR(µmol h^−1^ plant^−1^)	Nodule number	AR per nodule (nmol h^−1^)
		**5 weeks**			**6 weeks**		
RU2222	wild-type	3.3±0.1	502±31	6.6±0.8	4.5±0.4	751±30	6.0±0.3
RU1933	*aapJQM*::ΩSp*braE::*Tn*phoA*	2.0±0.2	347±15	5.5±0.9	2.2±0.3	369±46	6.1±0.4

The rates of acetylene reduction and numbers of nodules by plants inoculated with RU1933 were significantly different from those of plants inoculated with wild-type RU2222 (p = 0.01; n≥6). ND  =  not determined. P-values were determined by ttest.

At 5 and 6 weeks the reduced symbiotic performance of strain RU1933 (Table 5) can be explained by the reduced number of nodules alone. However, at 4 weeks the reduction in nitrogen fixation rates is only partly explained by the reduced nodule number (Table 4). The nitrogen fixation rate per nodule and bacteroid is reduced as well.

**Figure 2:**
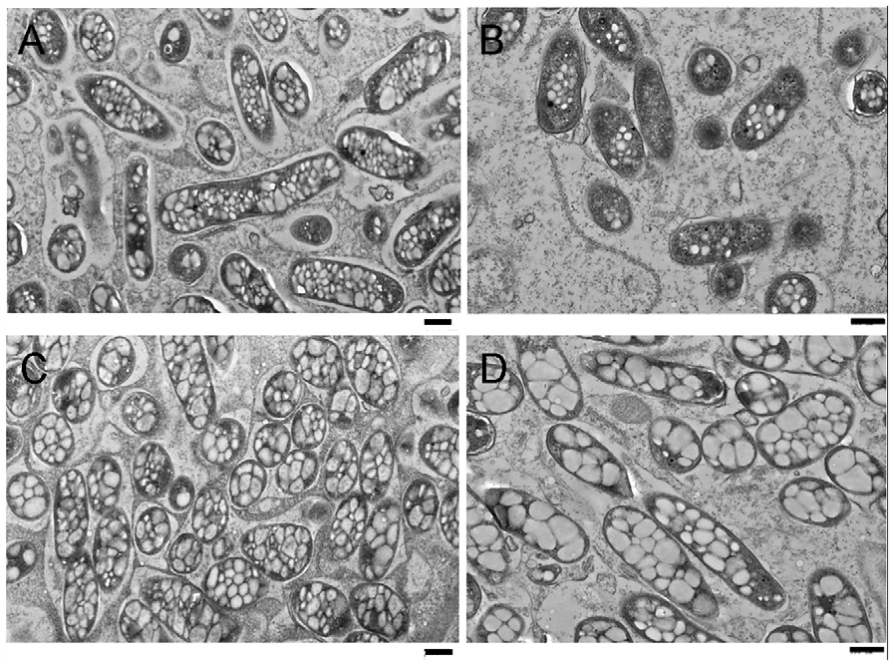
TEM pictures of bacteroids of wild-type (A and B) and RU1933 *aap bra* (C and D). A and C are pictures taken of 4 week old nodules, B and D of 6 week old nodules. Size bars are 500 µm.

TEM pictures of bacteroids formed by wild-type RU2222 and the *aap bra* mutant RU1933 showed remarkable differences (Figure 2). Bacteroids of determinate French bean nodules usually show an accumulation of granules of the carbon storage compound poly-hydroxybutyrate (PHB). This accumulation was enhanced in the *aap bra* mutant background RU1933 (Figure 2). TEM micrographs were prepared from all the three experiments harvested after 4, 5 and 6 weeks and always showed identical results.

### Properties of a *Sinorhizobium meliloti* 2011 *aap bra* transport mutant

*S. meliloti livHMGFK* is homologous to *R. leguminosarum braDEFGC* [13], so for clarity we hereafter name *S. meliloti livK* as *braC* and *livH* as *braD.* The double mutants *aapJ*::ΩSp *braC*::mTn*5* (LMB210) and *aapJ*::ΩSp *braD*::mTn*5* (LMB211) were generated in the *S. meliloti* 2011 background as described in Materials and Methods. All amino acid transport was reduced to background levels in the *aapJ braD* mutant LMB211, which is very similar to *R. leguminosarum* strains (Table 6). Thus mutations in the SBP of the Aap and integral membrane proteins of the Bra abolish most amino acid transport in *S. meliloti.* However, strain LMB210 which contains a mutation in the gene coding for the SBP BraC, still transported isoleucine at a high rate. As previously described for *R. leguminosarum* bv. *viciae* 3841 an orphan SBP (BraC3) is able to interact with the Bra membrane complex and is very specific for the branched-chain amino acids and alanine [2]. A possible orthologue (SMc00078) of BraC3 is present in *S. meliloti* strain1021.

**Table 6:** Transport of amino acids by strains of *Sinorhizobium meliloti.*

Strain	Genotype	Glutamate	Isoleucine	Leucine	Valine	Alanine
2011	wild-type	23.5±0.4	16.7±0.8	16.2	15.5	30.1
LMB210	*aapJ*::ΩSp*braC*::mTn*5*	0.8±0.1	12.0±0.6	ND	ND	ND
LMB211	*aapJ*::ΩSp*braD*::mTn*5*	0.6±0.1	0.7±0.3	<0.4	0.8±0.2	4.4±0.4

All strains were grown in M9 modified glucose/NH_4_Cl minimal medium. Rates of uptake are expressed as nmol min^−1^mg^−1^ protein. Values are the mean ± SEM of determinations from three independent cultures, except for strain 2011 alanine, leucine and valine transports, which just served as controls and represent single measurements. ND  =  not determined.

### Plant phenotypes of *S. meliloti* aap *bra* mutants on alfalfa

*Medicago sativa* plants inoculated with wild-type 2011 or the *aapJ braD* double mutant LMB211 were analysed for nitrogen fixation (AR) and dry weights after 4 weeks of growth. Neither nitrogen fixation rates nor dry weights differed significantly (p>0.05) between *S. meliloti* 2011 and LMB211, although uninoculated control plants showed strong signs of nitrogen starvation and a corresponding drop in dry weights (Table 7).

**Table 7:** Plant dry weights and acetylene reduction (AR) of alfalfa (*M. sativa*) at 4 weeks.

Strain	Genotype	Plant dry weight(mg plant^−1^)	AR(µmol h^−1^ plant^−1^)
2011	wild-type	66.8±7.7	0.33±0.05
LMB211	*aapJ*::ΩSp*braD*::miniTn*5*	64.9±6.4	0.46±0.04
uninoculated		36.5±2.2	ND

The values are the mean ± SEM of plants inoculated with 2011 (n = 18), LMB211 (n = 18) and uninoculated control plants (n = 6). ND  =  not determined.

TEM pictures of wild-type 2011 and the *aapJ braD* mutant LMB211 bacteroids showed no obvious differences in development (data not shown) and flow cytometric characterisation of bacteroids also failed to demonstrate developmental alterations of bacteroids formed by LMB211 (data not shown).

## Discussion

Pea plants inoculated with *Rhizobium leguminosarum* bv. *viciae* strains mutated in the two broad specificity amino acid ABC uptake systems, Aap and Bra develop a severe nitrogen starvation phenotype [1], [2]. However, the very broad solute specificity of Aap and Bra made it difficult to determine which amino acids are responsible for this phenotype. Therefore, *braC* was mutated in an *aap* null mutant background and it was shown that strain RU1979 (Δ*aapJ braC*::ΩSpec) retains the transport of branched-chain amino acids. This is because of the presence of an orphan SBP (BraC3) which specifically binds branched-chain amino acids and interacts with the core Bra membrane complex. Strain RU1979, which only retains branched-chain amino acid transport, fixes nitrogen at wild-type rates on pea plants. In addition complementation analysis using heterologous ABC transport systems demonstrated that branched-chain amino acid transport alone rescues the starvation phenotype on pea plants. Microarray analysis of pea bacteroids showed that the pathways of branched-chain amino acid biosynthesis are transcriptionally down regulated leading to premature arrest of development in *aap bra* bacteroids [2].

Our first aim in this study was to determine how much branched-chain amino acid transport is needed for fully effective N_2_-fixation. It was already known that the single *aap* and *bra* mutants have no symbiotic phenotype so the wild-type rate of transport can be lowered by ∼50% with no effect [1]. However, we had previously identified a region likely to be important in solute specificity in AapQ [12] and mutation of P144D reduced branch chain amino acid transport by ∼60%. The absolute rates of branched-chain amino acid transport in RU1976 (*aapQ* P144D *braEF*::ΩTc) were 1–2 nmol min^−1^ mg protein^−1^, while the *aap bra* double mutant strains have rates below 1 nmol min^−1^ mg protein^−1^. This rate for RU1976 is roughly 10% of the maximum rates of branched-chain amino acid uptake measured in wild-type [2], [13]. However, RU1976 provided enough branched-chain amino acids for developing bacteroids to achieve a wild-type rate of nitrogen fixation. This supports the hypothesis that *R. leguminosarum* bacteroids are symbiotic auxotrophs and do not require amino acids for catabolism, which would need a much higher rate of transport.

A *leuD* mutant of *R. leguminosarum* strain 3841 required leucine at 100 µM added to AMS glucose/NH_4_Cl cultures to overcome auxotrophy and restore a wild-type rate of growth. By comparison as sole nitrogen source 10 mM leucine was needed to obtain the same growth rate as NH_4_Cl.

Published microarray data shows transcriptional down regulation of branched-chain biosynthetic genes occurs in *S. meliloti* in symbiosis with alfalfa (*M. sativa*) and *Bradyrhizobium japonicum* with soybean (*Glycine max*) [15], [16], [17]. Therefore, the requirement for branched-chain amino acid transport was investigated in other legume-rhizobia interactions. The first interaction investigated is that between *R. leguminosarum* bv. *phaseoli* and French bean. This was done for two reasons. First, beans have determinate nodules, while peas have indeterminate nodules. Second, *R. leguminosarum* bv. *viciae* A34 and *R. leguminosarum* bv. *phaseoli* 4292 differ only in their Sym plasmid so the same *aap bra* mutations can be directly compared in pea and bean hosts.

French bean plants inoculated with RU1933 (*aap bra*) became nitrogen starved, indicated by reduced dry weight, nitrogen fixation, and nitrogen fixation per nodule as well as yellowing and increased PHB accumulation in bacteroids. Unexpectedly, they produced fewer nodules on beans, in contrast to the increase in nodule formation on peas by equivalent *aap bra* mutants. Legumes usually increase nodule numbers when inoculated with fix reduced strains. The reduction in French bean nodules inoculated with RU1933 might result from limitation of branched-chain amino acids during infection or very early in bacteroid development leading to reduced success in nodule formation. Therefore, while peas and beans have different nodulation responses to *aap bra* mutants their different patterns of nodule development probably alter the timing of amino acid availability and the development of symbiotic auxotrophy.

In addition to reduced numbers of nodules elicited by RU1933 bacteroids had more PHB granules as visually scored from TEM pictures of 4, 5 and 6 week old nodules. Increased levels of carbon storage compounds in nodules, usually starch in the plant and PHB in bacteroids, are a typical feature of non-fixing or under-performing strains [1], [18], [19]. The plant provides carbon and reductant for nitrogen fixation, but the bacteroids of RU1933 may not use it efficiently. This suggests that in addition to less successful infection, nitrogen fixation by bean bacteroids is limited and leads to nitrogen starvation in the host plant. This might reflect, as for pea nodules, a general high demand for branched-chain amino acids [2], which are energetically expensive to make and are the most abundant amino acids in bacterial proteomes [20]. However, when inoculated with *aap bra* mutants, beans are less severely nitrogen starved than peas. For example French beans had dry weights reduced by 24% while peas were reduced by 59%, close to uninoculated controls [1].

Similar to *R. leguminosarum* Rlv3841 [2], [21], branched-chain amino acid biosynthesis is down regulated in *S. meliloti* [15], [16], [22]. Thus an *aap bra* mutant was isolated in *S. meliloti* 2011 and its rates of branched-chain amino acid transport were below 1 nmol min^−1^ mg protein^−1^. However, there were no differences in dry weight or acetylene reduction of alfalfa plants inoculated with mutant or wild-type. Inspection of TEM micrographs of bacteroids as well as FAC analysis of their size and DNA content did not reveal any differences between wild-type and mutant. Either the decrease in transcription of the branched-chain amino acid biosynthetic genes in *S. meliloti* 2011 bacteroids is not severe enough to produce symbiotic auxotrophy or residual transport rates by *aap bra* mutants are sufficient to support bacteroid maturation and persistence. The ability to reduce branched-chain amino acid transport to a very low rate in *R. leguminosarum* bv. *viciae* without inducing symbiotic auxotrophy is consistent with this. If the concentrations of branched-chain amino acids in alfalfa nodules are slightly higher than peas or the reduction in transcription of the genes for their biosynthesis is delayed or less severe, then symbiotic auxotrophy would not occur.

This study demonstrates that symbiotic auxotrophy is a phenotype right on the edge where it can be pushed in one direction or the other by small changes in conditions or gene expression. Perhaps the more important aspect of this is not whether a particular *Rhizobium-*plant association shows symbiotic auxotrophy for amino acids but, that bacteroids may shut down many aspects of metabolism as long as they retain the ability to transport essential nutrients from host plants. This emphasises the exquisite co-evolution of *Rhizobium* and legumes with the metabolism of bacteroids being organelle like.

## Materials and Methods

### Bacterial growth and media

The bacterial strains, plasmids and primers used in this study are detailed in Table 8. *Rhizobium* strains were grown at 28°C in either Tryptone Yeast extract (TY) [23] or acid minimal salts medium (AMS) [24] with 10 mM D-glucose as carbon sources and 10 mM NH_4_Cl as nitrogen source. *S. meliloti* was grown in M9 medium with 1.25 mM CaCl_2_, 2.5 mM MgSO_4_, 0.1 ug/ml Biotin, 10 ug/ml CoCl_2_, 10 mM D-glucose and 10 mM NH_4_Cl. Antibiotics were used at the following concentrations (µg ml^−1^): streptomycin (St), 500; neomycin (Nm), 80; tetracycline (Tc), 5; gentamicin (Gm), 20 and spectinomycin (Sp), 100.

**Table 8:** Strains, plasmids, primers.

Strains, plasmids, primers	Description	Reference, source
Strain		
3841	*Rhizobium leguminosarum* bv. *viciae*; Str^r^	[33]
RU1721	*braEF*::ΩTc	This study
RU1976	*braEF*::ΩTc *aapQ* P144D	This study
RU1722	*aapJQM*::ΩSp *braEF*::ΩTc	[1]
4292	Sym plasmid pRL2JI (*phaseoli*) derivative of 8401 bv. *phaseoli*, Str^r^	[34]
RU2222	4292 with pLAFR1	This study
RU1809	*aapJQM*::ΩSp with pLAFR1	This study
RU1932	*braE*::Tn*phoA* with pLAFR1	This study
RU1933	*aapJQM*::ΩSp *braE*::Tn*phoA* with pLAFR1	This study
2011	2011, Str^r^ derivative of *Sinorhizobium meliloti*	J. Dénarié, France
2011mTn5STM.3.12.F11	*livK*(*braC*)::mTn*5*	[27]
2011mTn5STM.4.05.D04	*livH*(*braD*)::mTn*5*	[27]
LMB210	*aapJ*::ΩSp *livK*(*braC*)::mTn*5*	This study
LMB211	*aapJ*::ΩSp *livH*(*braD*)::mTn*5*	This study
Plasmid		
pLAFR1	Wide-host-range mobilizable P-group cloning vector; Tc^r^	[35]
pJQ200SK	pACYC derivative, P15A origin of replication; Gm^r^	[36]
pPH1JI	P-group chaser plasmid	[37]
pRU1247	*aapJQMP* region with AapQ P144D mutation on pJQ200SK	This study
pRU410	*aapJQM*::ΩSp on pJQ200SK	[25]
pBIO206	*braE*::Tn*phoA* on pLAFR1	[25]
pJET1.2	Cloning vector	Fermentas, Lithuania
pLMB111	Sm2011 2.6 kb *aapJ* fragment on pJET1.2	This study
pLMB112	*aapJ* deletion with *Bam*HI site in pLMB111	This study
pLMB113	*XbaI/XhoI* fragment of pLMB112 cloned into pJQ200SK	This study
pLMB118	ΩSp insertion into *Bam*HI site of pLMB113	This study
Primer		
p495	CACCACTAGTCATAGACCGAATCGACCAC	
p441	AAACTAGTGGGTCTTTTCAAAGGATA	
p418	GGGTCGCGAAACCAGACTTT	
p419	CGCCGTACCACTTGTTCATG	
pIS50R	AGGTCACATGGAAGTCAGATC	
p439	ATAGGGGCCGACACCGATGA	
pr0163	GATTGCCGGAATATCCTGCA	
pr0164	GCGGCAGACCGATGATCAGC	
pr0165	TTTGGATCCTTTTTTGAACCTTTTCGGTT	
pr0166	TTTGGATCCTGATCGGACGCTATGTCGGG	
pOTforward	CGGTTTACAAGCATAAAGC	
pr0240	GCACGTGCGTCCCGCCGATC	
pr0241	ACGATCTCGATCGACTGTCC	

Str  =  streptinomycin; Tc  =  tetracycline; Sp  =  spectinomycin; Gm  =  gentamicin; *Bam*HI sites in primers are underlined.

### Isolation of mutants

The *Rhizobium leguminosarum* bv. *viciae* AapQ site directed mutant was generated as follows. *AapJQMP* was amplified using primers p495 and p441. The 5 kb PCR product was TOPO cloned into pENTR/D-Topo (Invitrogen, USA) and the CCG triplet coding for proline at position 144 within AapQ replaced by a GAC coding for aspartic acid (P144D) using the Quick Change site-directed mutagenesis kit (Stratagene, USA). The region was transferred by a Gateway LR reaction into the pJQ200SK derivative, pGW1, producing pRU1247. This plasmid was conjugated into RU1722 (*aapJQM*::ΩSp *braEF*::ΩTc) to generate the homogenote RU1976 by sucrose selection and loss of the spectinomycin marker. The correct mutation was confirmed by PCR.

*Rhizobium leguminosarum* bv. *phaseoli* mutants were generated as follows. Plasmid pRU410 [25], which contains an omega spectinomycin (ΩSp) cassette cloned into an *Mfe*I deletion (3.2 kb) spanning *aapJQM,* was transformed into *Escherichia coli* S17-1 and conjugated into *R. leguminosarum* bv. *phaseoli* 4292. This plasmid is derived from pJQ200SK [26] which contains the *sac* genes. Transconjugants were spread on AMS agar plates [24] containing sucrose (10%) as a carbon source and spectinomycin resistant, gentamicin sensitive colonies were selected. One colony (RU1809) was confirmed to have the correct *aapJQM*::ΩSp genotype by PCR mapping using primers p418 and p419. As expected from the phenotype of *aap* mutants of *R. leguminosarum* bv. *viciae*, strain RU1809 did not grow on minimal medium with L-glutamate as the sole carbon and nitrogen source. To generate *bra* mutants, cosmid pBIO206A [25] (pIJ1427 *braE*::Tn*phoA*) was transformed into S17-1 and conjugated into strain 4292 and RU1809. Colonies were selected for tetracycline and kanamycin, resistance, before the chaser plasmid pPH1JI (gentamicin resistant) was then conjugated into them to allow the selection of recombinants with loss of the tetracycline marker and resistance to kanamycin and gentamicin. Plasmid pPH1JI was then removed by conjugating pLAFR1 into all strains, and selecting for loss of the gentamicin marker while retaining resistance to kanamycin and tetracycline. The *braE*::Tn*phoA* insertion was confirmed by PCR mapping using primers pIS50R and p439. The *braE*::Tn*phoA* single and *aapJQM*::ΩSp *braE*::Tn*phoA* double mutants were designated as RU1932 and RU1933, respectively. Strain 4292 containing pLAFR1 was designated RU2222.

*Sinorhizobium meliloti* mutants were generated as follows. A series of mTn5STM mutants in the *livHMGFK* operon of *S. meliloti* strain 2011 [27] were obtained from Anke Becker at CeBiTec, Bielefeld, Germany. The *S. meliloti livHMGFK* is homologues to the *R. leguminosarum braDEFGC* operon [13]. A *livK* (*braC*) mutant 2011mTn5STM.3.12.F11 and a *livH* (*braD*) mutant 2011mTn5STM.4.05.D04 were chosen as a background for an *aapJ* insertion mutation using an ΩSp cassette [28]. Primers pr0163 and pr0164 were used to amplify a 2.6 kb region surrounding the *S. meliloti* strain 2011 *aapJ* gene. The resulting PCR product was cloned into pJET1.2 (Fermentas, Lithuania) resulting in plasmid pLMB111. Primers pr0165 and pr0166 were used in an inverse PCR to amplify the *aapJ* surrounding DNA from pLMB111 without the *aapJ* ORF. The resulting PCR product was digested with *Bam*HI (underlined in primers) and religated forming plasmid pLMB112. An *Xba*I/*Xho*I fragment was then cloned from pLMB112 into pJQ200SK [26] to generate pLMB113. This vector was opened with *Bam*HI and a ΩSp cassette was inserted producing pLMB118. This plasmid was used to generate *aapJ*::ΩSp homogenotes in the *braC* and *braD* mutant backgrounds to produce strains LMB210 and LMB211, respectively. The insertion was mapped from both sides using primers pOTforward and pr0240 or primer pr0241, respectively.

### Transport assays

*R. leguminosarum* uptake assays were performed with 25 µM (4.625 kBq of ^14^C) solute [13], using cultures grown in AMS with 10 mM D-glucose and 10 mM NH_4_Cl to an OD_600_ of ∼0.4. *S. meliloti* was grown in modified M9 medium with 10 mM D-glucose and 10 mM NH_4_Cl to an OD_600_ of ∼0.4.

### Plant assays

French bean plants (*Phaseolus vulgaris* cv. Tendergreen) were surface sterilised and grown for 5 to 6 weeks in a sterile mixture of fine gravel and vermiculite (1∶4) in 15 l pots with 7 seeds per pot in a controlled environment at 22°C and a 8:16 h day: night cycle as described earlier [29]. The 4 week experiments were performed in 8 l pots in a fine gravel and vermiculite mixture (1∶4) with 7 seeds per pot in a controlled environment at 22°C and a 8:16 h day: night cycle. Pots were open to drain into a sterile autoclave bag and watered with nitrogen free rooting solution [30]. *Medicago sativa* cv. Europe plants were grown in 1 l pots in vermiculite with 7 seeds per pot for 4 weeks watered with nitrogen free rooting solution [30].

Acetylene reduction was measured as previously described [31].

Bean shoot dry weights were determined after 8 weeks, pea shoot dry weights after 6 weeks and alfalfa shoot dry weights after 4 weeks of growth.

### Bacteroid assays

After counting and removing all nodules from the plants, nodules were crushed in 40 mM HEPES buffer and bacteroids harvested by differential centrifugation [32]. Bacteroid pellets were then broken in a FastPrep FP120 ribolyser (Bio101, Thermo) and protein levels determined following a standard Bradford assay.

Flow cytometric analysis of *S. meliloti* bacteroids was performed as described elsewhere [2].
